# Impact of Gender Difference on Anxiety in COVID-19 Patients in Quarantine Wards

**DOI:** 10.3389/fpsyt.2021.799879

**Published:** 2022-01-21

**Authors:** Peng Sun, Tao He, Duo-jiao Fan, Chao Xu, Ying Wang, Shuang-wei Song, Feng Jin, Xiao-xue Chen, Mei Yang

**Affiliations:** ^1^Department of Emergency Medicine, Baoding Second Hospital, Baoding, China; ^2^Department of Tuberculosis, Tangshan Infectious Disease Hospital, Tangshan, China; ^3^Department of Science and Education, Baoding Second Hospital, Baoding, China; ^4^Department of Otolaryngology, Hospital of the Joint Logistic Support Force of the People's Liberation Army, Tangshan, China; ^5^Department of Surgical Oncology, Baoding Second Hospital, Baoding, China; ^6^Department of Respiratory, Dingzhou City People's Hospital, Baoding, China

**Keywords:** novel coronavirus pneumonia, anxiety, mood, gender difference, quarantine ward

## Abstract

**Objective:**

This study aimed to investigate the gender difference in anxiety in novel coronavirus pneumonia (COVID-19) patients in the quarantine ward during the outbreak.

**Methods:**

The self-rating anxiety scale (SAS) was used on the seventh day of isolation to analyze the anxiety levels of a total of 242 suspected or confirmed COVID-19 patients in the quarantine wards of two hospitals; 232 of these patients (112 males and 120 females) completed the anxiety scoring. The anxiety scores were compared between male and female patients using the *t*-test, and a scatter diagram was used for analysis.

**Results:**

The SAS scores of females in quarantine wards were higher than those of males at seven days of isolation.

**Conclusion:**

Women with COVID-19 are more prone to anxiety than men while in isolation. It is necessary to give more attention to female patients in quarantine wards in the future, and psychological counseling may be necessary.

## Introduction

An outbreak of the novel coronavirus occurred in China in December 2019. China is a country with a large population, and the epidemic is a challenge for public health. A study revealed that the latent period of novel coronavirus pneumonia is 1–14 days and can be up to 24 days. This indicates that both infected and suspected patients need to be isolated for a long time. During the epidemic isolation period, the psychological anxiety of those patients in isolation for a long time may be very serious for both sexes; however, women may be less likely to tolerate excessive psychological pressure. Anxiety disorder is a neurosis characterized by paroxysmal or persistent emotional anxiety and tension. In recent years, the incidence of anxiety disorders in China has been increasing; anxiety and depression now rate highest in the total burden of diseases in China, accounting for around 20%, and seriously threatening people's health (1, 2). As a result of the COVID-19 outbreak, women are experiencing higher levels of psychological distress than men (2, 3).

The purpose of this study is to evaluate whether gender factors have an impact on the anxiety state of isolated patients, and to provide a reference for clinical practice.

## Methods

The self-rating anxiety scale (SAS) (2, 3) was used on the 17th day of isolation to analyze the anxiety levels of 242 patients, 69 of which were suspected COVID-19 patients in the quarantine wards of the Infectious Diseases Department of Baoding Second Hospital and 173 were suspected or confirmed COVID-19 patients in Tangshan City Infectious Disease Hospital. The date range for the study is from January 24th to April 9th, 2019. One patient with severe infection (in Tangshan City Infectious Disease Hospital) died, and nine children were unable to complete the SAS; the remaining 232 patients (112 males and 120 females) completed the anxiety scoring at 7 days of isolation. There were 145 patients who lost follow-up when we collected the scales. In the end, 145 lost follow-up when we collected the scales, and 97 eventually completed the follow-up.

Data were statistically analyzed using statistical software SPSS17.0. All results were expressed as mean ± standard deviation (x ± SD). Intergroup comparison was conducted using the *t*-test. Scatter diagram was devolopped to demonstrate the relationship between gender and SAS scors. A *P*-value of < 0.05 was considered statistically significant.

## Results

**Figure 1:**
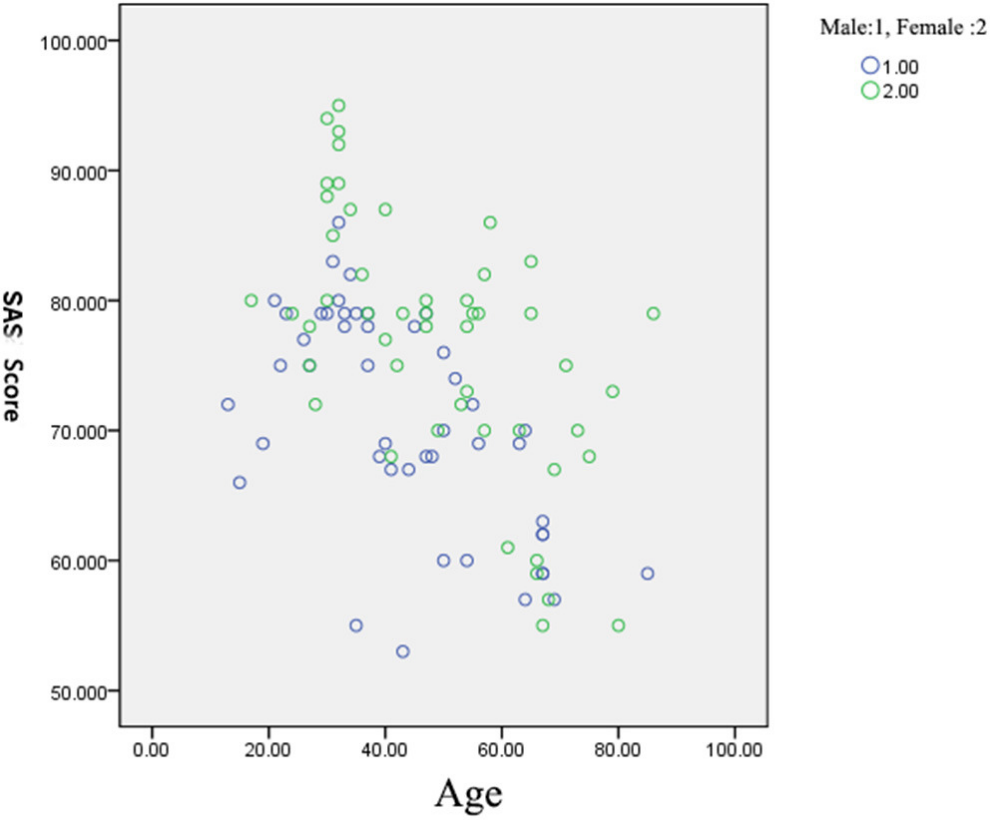
Impact of gender on SAS score.

A total of 232 patients participated in the anxiety scoring, and the anxiety scores of male and female patients were compared. The 97 patients completing final follow-up included 47 males and 50 females. The mean age of male patients were 43.45 ± 16.64 years, ranging from 13 to 85 years. The mean age of female patients were 49.14 ± 17.33 years, ranging from 17 to 86 years. The results revealed that women had higher anxiety scores than men (Table 1). This suggests that women are more prone to anxiety than men during isolation. The scatter diagram (Figure 1) reflects the correlation and demonstrates that the SAS score of females were higher than those of males, indicating that women are more likely to have anxiety in quarantine wards than men.

**Table 1:** Influence of gender on SAS scores.

**Gender**	**Number of case (n)**	**SAS Score ($\bar{\text{X}}$ ±s)**
Male	47	70.638 ± 8.515^*^
Female	50	76.800 ± 9.889^*^

* *p < 0.01 Male vs Female*.

## Discussion

In 1992, the Institute of Medicine published a report on emerging infections, which were bringing more and more challenges to public health (3). This paper explained that people should attach great importance to the risk of infectious diseases, and it even used an “alarm” as a warning. Medical development and scientific progress during the second half of the twentieth century aimed to reverse the earlier trend of complacency toward infectious disease risk (4). In March 2003, the International Organization for Migration published an article by James M. Hughes, M.D. (the director of the National Center for Infectious Diseases, Disease Control and Prevention) that described the challenges of infectious diseases to human beings and emphasized the need for more countries to recognize and respond to the threat caused by infectious diseases. In the past few years, infectious disease pathogens have emerged at an alarming rate. Recent examples include West Nile virus, monkey smallpox, and anthrax in the United States (5–9). Currently, all over the world, the isolation of COVID-19-positive patients is widely used to prevent and deal with the epidemic. However, while many scholars have conducted a great deal of research on the prevention and treatment of the epidemic, few studies have examined the anxiety of isolated patients (10–13).

Epidemiological investigation of COVID-19 has revealed that we should direct attention to both the prevention of hospital infection and to psychological intervention as well as the prevention of mental health problems in patients, survivors, isolated people, volunteers and above (14–18). The significance of this study lies in the fact that during the epidemic isolation period, the psychological anxiety of those patients in isolation for a long time is likely to be very serious; in particular, female patients in isolation should be given more attention by medical staff in order to avoid suicide, medical injury, and other adverse events (19–22).

Because of the timeframe and the hospital level, only 97 cases were analyzed in this study, all the patients were over 18 years old; in the future, more cases will be analyzed and summarized. This study has scientific significance for the early detection and psychological intervention for anxiety in isolated patients. It provides a strong basis for the necessity of identification and treatment of the psychological anxiety of patients who are isolated, both during the COVID-19 epidemic now and for epidemics in the future.

## Data Availability Statement

The original contributions presented in the study are included in the article/supplementary material, further inquiries can be directed to the corresponding author.

## Ethics Statement

The studies involving human participants were reviewed and approved by Baoding Second Hospital. The patients/participants provided their written informed consent to participate in this study.

## Author Contributions

PS and TH: conceptualized and designed the study, drafted the initial manuscript, and reviewed and revised the manuscript. CX, YW, S-wS, and FJ: designed the data collection instruments, collected data, carried out the initial analyses, and reviewed and revised the manuscript. D-jF: coordinated and supervised data collection and critically reviewed the manuscript for important intellectual content. All authors approved the final manuscript as submitted and agree to be accountable for all aspects of the work.

## Conflict of Interest

The authors declare that the research was conducted in the absence of any commercial or financial relationships that could be construed as a potential conflict of interest.

## Publisher's Note

All claims expressed in this article are solely those of the authors and do not necessarily represent those of their affiliated organizations, or those of the publisher, the editors and the reviewers. Any product that may be evaluated in this article, or claim that may be made by its manufacturer, is not guaranteed or endorsed by the publisher.
